# A Rigid Image Registration Based on the Nonsubsampled Contourlet Transform and Genetic Algorithms

**DOI:** 10.3390/s100908553

**Published:** 2010-09-14

**Authors:** Fatiha Meskine, Miloud Chikr El Mezouar, Nasreddine Taleb

**Affiliations:** 1 Department of Electrotechnics, University of Mascara, Mascara 29000, Algeria; 2 RCAM Laboratory, College of Engineering, Djillali Liabes University, Sidi-Bel-Abbes 22000, Algeria; E-Mails: chikrelmezouar@univ-sba.dz (M.C.E.M.); ne_taleb@univ-sba.dz (N.T.)

**Keywords:** genetic algorithms, image registration, multi-resolution analysis, nonsubsampled contourlet transform, wavelet transform

## Abstract

Image registration is a fundamental task used in image processing to match two or more images taken at different times, from different sensors or from different viewpoints. The objective is to find in a huge search space of geometric transformations, an acceptable accurate solution in a reasonable time to provide better registered images. Exhaustive search is computationally expensive and the computational cost increases exponentially with the number of transformation parameters and the size of the data set. In this work, we present an efficient image registration algorithm that uses genetic algorithms within a multi-resolution framework based on the Non-Subsampled Contourlet Transform (NSCT). An adaptable genetic algorithm for registration is adopted in order to minimize the search space. This approach is used within a hybrid scheme applying the two techniques fitness sharing and elitism. Two NSCT based methods are proposed for registration. A comparative study is established between these methods and a wavelet based one. Because the NSCT is a shift-invariant multidirectional transform, the second method is adopted for its search speeding up property. Simulation results clearly show that both proposed techniques are really promising methods for image registration compared to the wavelet approach, while the second technique has led to the best performance results of all. Moreover, to demonstrate the effectiveness of these methods, these registration techniques have been successfully applied to register SPOT, IKONOS and Synthetic Aperture Radar (SAR) images. The algorithm has been shown to work perfectly well for multi-temporal satellite images as well, even in the presence of noise.

## 1. Introduction

Image registration is the process of overlying two or more images of the same scene taken at different times, from different viewpoints and by different sensors [1]. The major purpose of registration is to remove or suppress geometric distortions between the reference and sensed images, which were introduced due to different imaging conditions, and thus to bring images into geometric alignment. Image registration is a crucial step in all image analysis tasks in which the final transformation is obtained by combining various data sources [2]. Typically, registration is required in remote sensing [3], as in multispectral classification, environmental monitoring, image fusion, change detection and weather forecasting, in medicine [4–7], as in combing computed tomography (CT) and nuclear magnetic resonance (NMR) data to obtain more complete information about the patient, monitoring of tumor growth, treatment verification, and in computer vision, as to target localization and automatic quality control.

Image registration can be defined as a determination of one-to-one mapping between the coordinates in one image space and those in another, such that points in two image spaces that correspond to the same scene point are mapped to each other. In case of image to scene registration, image coordinates are mapped to the corresponding points in the scene. As an example of a 2D image, for a rigid-body transformation, the transformation parameters *T_p_* in (1) consist of three parameters, two shifts (*t_x_,t_y_*) and a rotation *θ*.

(1) $$T_{P}\;(x,y)=\left(\begin{array}{cc} \text{cos}\;\theta & \text{sin}\;\theta \\ -\text{sin}\;\theta & \text{cos}\;\theta \end{array}\right)\left(\begin{array}{c} x\\ y \end{array}\right)+\left(\begin{array}{c} t_{x}\\ t_{y} \end{array}\right)$$

Thus the registration problem is to find the optimal spatial transformation that matches the images, either for the purpose of determining the parameters of matching transformation or to expose the differences between the images. Image registration problems typically consist of three major components [2]: (i) the transformation space that determines the allowed spatial transformation applied to images. This component is highly application dependent. Examples are rigid-body, affine and deformable transformations. (ii) The registration function quantifies the similarity between two images under a given transformation. Some examples are mutual information, correlation ratio, sum of absolute difference, *etc.* (iii) The optimization method searches for the optimum transformation that maximizes the similarity between the images.

Genetic algorithms (GAs) [8,9] are optimization methods that mimic the process of evolution which have received much attention. GAs have been widely applied to different optimization problems due to their robustness. They have been often successful in dealing with most multi-modal and complex problems. The main specific of the GA as an optimization method is their implicit parallelism, which is a result of the evolution and hereditary process. A GA is, in fact, a driven stochastic search technique, which combines stochastic (represented by mutation operator) and “logical” search (represented by crossover of parental chromosomes and survival of the fittest by appropriate selection). These characteristics of GAs offer possibilities for their improvement by appropriate balance between exploration and exploitation.

Image registration can take advantage of the robustness of GAs in finding the optimum transformation. Most GA applications have been hybrids [10]. This happens because there is a possibility of incorporating domain knowledge which gives it an advantage over a pure blind search. Hybridization of GAs has been done on a range of image registration applications [11,12]. This technique has been proved to provide fast convergence and good performance in finding correct registrations compared to a GA alone. The hybrid method of cooperating GA with elitism and fitness sharing techniques is proposed in this paper to maximise the robustness of the search technique in order to provide better quality in image registration. Moreover, this approach has been used within a multi-resolution framework based on the nonsubsampled contourlet transform.

The reminder of this paper is organized as follows: Section 2 presents a review of previous works in image registration using wavelet and genetic algorithms. The third topic focuses on the multi-resolution transforms as the wavelet decomposition and nonsubsampled contourlet transform. An overview of GAs, and hybrid techniques adopted in this work are presented in Section 4. The fifth topic describes the components of GAs used for registration. Section 6 introduces the proposed methods of registration based on the nonsubsampled contourlet transform. In the next section, the results of simulation are illustrated, followed by a conclusion.

## 2. Related Work

The wavelets transform is a powerful tool for multi-resolution analysis. When using such multi-resolution data, the size of the search data can be reduced by initially searching at lowest resolution and then proceeding to higher resolution images where the search results are only refined [13–15].

Most of the previous work in image registration has focused on the use of wavelets. LeMoigne *et al.* presented a cross-comparison of automated registration algorithms for multiple source remote sensing data in [16], in which a multi-resolution wavelet-based (MRW) image registration was used. The algorithm requires no *a priori* knowledge in order to perform automatic registration. The similarity measure is based on the normalized cross-correlation. The work in [17] proposed automatic wavelet-based image registration based on point matching techniques. Unlike LeMoigne’s technique, the similarity measure is based on automatically extracted control points from the wavelet-compressed images. The registration result is determined by matching these control points. However, using similar test data as in [16], poor registration accuracy results were reported. The translation invariant wavelets and their application were explored in [18]. An image registration algorithm using translation invariant wavelets was developed. However, the study produced poor results and concluded that the translation invariant wavelets have limited applications in image registration. Fonseca and Manjunath presented a multi-resolution registration that relies on the grey level information content of the images and their local wavelet transform modulas maxima [19]. The proposed algorithm consists of five major steps: (1) smoothing the image, (2) decomposing the image using wavelets, (3) extracting the feature points, (4) matching the feature points, and (5) refining the matching in higher resolutions. The registration error is less than one pixel for noise-free images. However, only clean images were used. The registration might fail if the images contain anomalies. The work in [20] investigates the use of wavelets to automatically register remotely sensed images. The proposed algorithm is based on the Laplacian of Gaussian filter to automatically extract ground control points and the discrete wavelet transform for multi-resolution analysis. The inherent multi-resolution processing of the image data provides an efficient method for registering large image data sets.

Image registration can be regarded as an optimization problem, where the goal is to find the best transformation parameters which maximize the measure similarity between compared images. GAs have been known to be a robust technique for search and optimization problems. Unlike traditional linear searches, GAs adaptively explore the search solution space in a hyper-dimensional fashion so that they can improve computational efficiency. Numerous researchers have attempted to apply GAs to help search over the complex search landscape in image registration.

Fitzpatrick was one of the first to investigate the applicability of GAs for image registration [21]. His work focuses on medical images obtained by X-ray, gamma ray, and imaging (NMR). Experiments were carried out using simulated data, however neither accuracy nor computing performance was quantified. In order to improve the performance, Ozkan [22] proposed a parallel implementation of Fitzpatrick’s GA but no algorithmic analysis or performance evaluation was quantified. Dasgupta and McGregor [23] proposed a structured GA for automatic registration organized in two levels: the higher level activates or deactivates sets of lower level genes. Although this GA is claimed to be five times faster than the Fitzpatrick algorithm, neither quantative accuracy measurements nor quantitative computing performance were presented. Turton and Arslan reported a hardware VLSI based design of their parallel GA registration [24]. The image registration is done in a compressed domain using the discrete cosine transform. The coefficients found under transformation have some limitations in their implementation. The work in [25] proposes a high speed image registration algorithm that determines the frame-to frame translational motion in an image sequence. A modified GA and the sequential similarity detection algorithm are used to achieve fast registration. The problem with this method is that the chromosome length is very limited and similarity measure is not as good as the normalized cross-correlation. Maslov and Gertner [26] proposed to include a gradient analysis of the fitness function within the GA iteration, in order to better drive the search space exploration. Experimentation shows that this approach can increase the efficiency of GAs when they are applied to an image registration problem. Laksanapanai [27] proposed a parallel implementation of GA based intensity image registration using MPI (Massage Passing Interface). The application field is medical image registration. The result for multi-modality alignment is very promising.

All these works have attempted to apply either wavelets or GAs, but fewer works have combined these two approaches in one technique, as those of Chalermwat and Ghazawi [28,29], who utilize the multi-resolution property in a wavelet domain and GA to reduce the search data size as well as the search scope. Using a coarse to fine grain scheme, multi-resolution techniques reduce the size of the search data by searching initially at lowest resolution first and then proceed to higher resolution where the search results are only refined. For each level, the best result found from the previous level is used as a center of the search. This procedure is called multi-resolution Iterative Refinement Algorithm (IRA). Finally at full resolution, the GA based registration is performed on windowed images from the reference and input images. The algorithm reports the registration results in terms of rotation and x-y axis translation.

Recently, new multi-resolution approaches have been developed in image processing like the contourlet transform and its shift invariant transform, namely the nonsubsampled contourlet transform (NSCT). In [30], Serief *et al.* have proposed a new technique using the NSCT transform to primarily extract feature points for image registration. In this paper, we have oriented our work to apply the NSCT multi-resolution decomposed images in order to reduce the search space in combination with GAs for the purpose of search optimization.

## 3. Multi-Resolution Transforms

### 3.1. Wavelet Transform

The multi-resolution scheme developed by Mallat provides a very fast algorithm which increases the importance of wavelets for on-line processing of imagery data. Wavelet based multi-resolution preserves most of the important features of the original data even at a low resolution. It also eliminates weak features in higher resolution, while highlighting strong image features [31].

The ordinary wavelet transform consists of filtering and downsampling operations. The necessary two-dimensional filtering operations are implemented via separable filters. At each level of the wavelet decomposition, four new images are created from the original image. The new images are named according to the filter (low-pass or high-pass) which is applied to the original image in horizontal and vertical directions. For example, the LH image is a result of applying the low-pass filter in horizontal direction and high-pass filter in vertical direction. Thus, the four images produced from each decomposition level are LL, LH, HL, and HH. The LL image is considered a reduced version of the original as it retains most details. The LH image contains horizontal edge features, and the HL contains vertical edge features, while the HH sub-band corresponds to the diagonal edges. Only the LL image is used to produce the next level of decomposition.

The standard two-dimensional wavelet transform is widely used in image processing. However, this technique fails to capture efficiently phenomena in images in directions other than the horizontal and vertical. Recently Do and Vetterli proposed an efficient directional multi-resolution image representation called the contourlet transform [32]. The contourlet transform is an extension of the Cartesian wavelet transform in two dimensions using multiscale and directional filter banks (DFBs), which offers a multi-resolution and directional decomposition. The transform employs the Laplacian pyramids to achieve multi-resolution decomposition and DFBs to achieve directional decomposition.

### 3.2. Nonsubsampled Contourlet Transform

Due to downsampling and upsampling, the contourlet transform is shift-variant. However, shift-invariance is desirable in image analysis applications such as edge detection, contour characterization, and image enhancement [33]. To overcome this problem, a shift-invariance version of the contourlet transform is proposed by [34] named nonsubsampled contourlet transform (NSCT). The NSCT is a shift-invariant version of the contourlet transform.

The NSCT is built upon iterated nonsubsampled filter banks to obtain a shift-invariant directional multi-resolution image representation. Unlike separable transforms such as wavelets, the NSCT can efficiently capture the intrinsic geometric structures in natural images such as smooth contour edges and is fully shift-invariant, multiscale and multidirection expansion that has a fast implementation.

**Figure 1.**
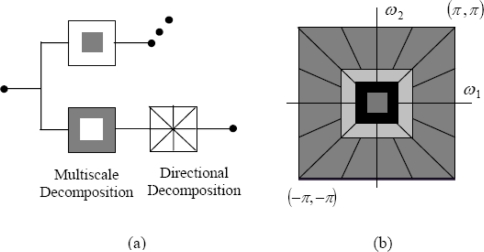
The nonsubsampled contourlet transform: (a) Block diagram. (b) Resulting frequency division, where the number of directions is increased with frequency.

The NSCT combines the nonsubsampled pyramids which provide multiscale decomposition and nonsubsampled DFB’s which provide directional decomposition (Figure 1). First a nonsubsampled pyramid split the input into a lowpass subband and a highpass subband. Then a nonsub-sampled DBF decomposes the highpass subband into several directional subbands. The scheme is iterated repeatedly on the lowpass subband outputs of the nonsubsampled pyramids. A nonsubsampled filter bank has no downsampling or upsampling, and hence it is shift-invariant.

## 4. Genetic Algorithms: Overview and Hybrid Techniques

### 4.1. Overview of Genetic Algorithms

A GA is a global stochastic search and optimization technique that can explore a large solution space and concentrate the search in the regions which lead to fitter structures, and hence better solutions of the problem [9].

GAs are iterative procedures that maintain a population of candidate solutions encoded in form of a chromosome string. According to evolutionary theory, only the chromosomes which have good fitness are likely to survive, generate offspring and pass on their strength by the genetic operators. The fitness of a chromosome is the way it is linked to the predefined problem or objective function. The evaluated candidates (chromosomes) will be selected for the reproduction in the next generation based on their fitness values. The two main operators used in the reproduction process are crossover and mutation. The purpose of those operations is to modify the chosen solutions and select the most appropriate offspring to pass on the succeeding generation until no better fitted solutions are possible. The crossover operator exchanges portions of the bit string hopefully to produce better candidates with higher fitness for the next generation. The mutation is then applied to perturb the string of chromosomes in to better explore the uncovered search space. Then the whole population is evaluated again in the next generation and the process continues until it reaches the termination criteria which can be triggered by finding an acceptable approximate solution or reaching a specific number of generations or until the solutions converge.

### 4.2. Hybrid Techniques

#### 4.2.1. Fitness Sharing Technique

The simple GA is able to explore effectively a multimodal search space. However it tends to converge to local optima when the search domain contains some local or global maxima (*i.e.*, in a multi-modal problem). This problem is the result of *genetic drift* which is a tendency of GAs to select a population with similar chromosomes, thus to converge towards one solution. Niching methods encourage GAs to explore more search space by maintaining genes’ diversity in the population and thereby converge to the global optima [35]. Niching methods have been developed to minimize the effect of genetic drift resulting from the selection operator in the traditional GA in order to allow the parallel investigation of many solutions in the population [36]. Several methods have been proposed, the most successful mechanism are *fitness sharing* and *crowding.*

In this work, we have employed the sharing technique. The idea behind the sharing method is to reduce the fitness of individuals that are very similar in their chromosome. The more individuals are located in the neighborhood of a certain individual, the more its fitness value is degraded. Mathematically, the shared fitness *f′_í_* of individual *i* with fitness *f_i_* is defined as follows:

(2) fi′=fi∑j=1Nsh(dij)

(3) With sh (dij) ={1−(dijσs)α if dij < σs0otherwise

where N denotes the population size and *d_ij_* represents the distance between the individual *i* and individual *j*. the sharing function (*sh*) measures the similarity level between two population elements according to a threshold of dissimilarity *σ_s_*. *α* is a constant parameter which regulates the shape of the sharing function (typically *α* = 1). The effect of this scheme is to encourage the search process in unexplored regions.

#### 4.2.2. Elitism Technique

**Figure 2.**
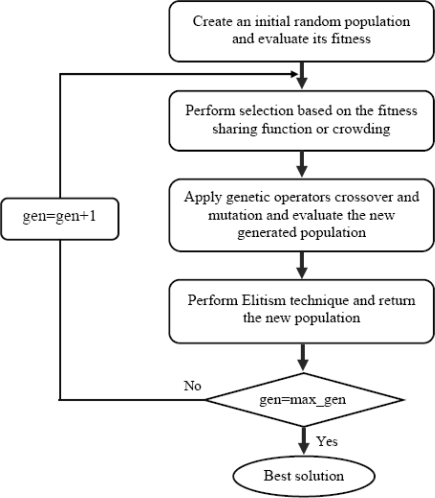
A proposed hybrid GA Scheme.

In basic genetic algorithms, it is possible for the next generation to have a best individual with a lower fitness than the best individual encountered in a preceding generation. This loss of the best individual occurs due to the probabilistic nature of the GA selection, crossover and mutation, and hence helps to improve the overall performance of the algorithm. To overcome this problem, we use the elitism technique. It is an effective tool to improve the performance capability of GAs, because it prevents losing the best found solutions by conserving the best solutions obtained in the optimization process. In this work, the best 5 percent of individuals in the population are preserved and copied in the next generation. The remaining are from the top ranked individuals after all the GA operations are performed. Figure 2 shows the details of the proposed hybrid GA scheme.

## 5. Genetic Algorithms Based Image Registration

Typically, a GA is composed of two main components, which are problem dependent: the encoding problem and the evaluation function.

### 5.1. Chromosome Encoding

**Figure 3.**
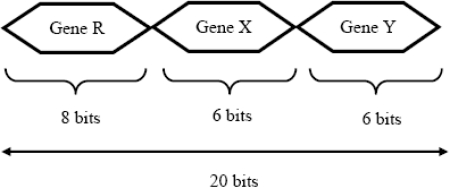
Chromosome encoding scheme.

In this paper, for 256 × 256 images, a binary string is adopted to represent a chromosome for rigid transformation. The chromosome string is composed of three genes. The gene R represents the rotational transform, the gene X represents the x-axis translational transform, and gene Y represents the y-axis translational transform as shown in Figure 3.

An 8-bit field is used to represent the possible relative rotation of the input image to the reference image; and 6 bits are used to express the translation in the x-axis and the y-axis. Thus the length of each chromosome is 20 bits. All representations are signed magnitude, using one bit for the sign and the rest of the bits to represent the magnitude of the rotation or translation. Thus, the relative rotation has the range of ±128 degrees, while relative translation in the x (or y) direction has the range of ±32 pixels.

### 5.2. Objective Function

To measure optimality, a fitness function can be used. This fitness function provides a numerical measure of the goodness of a proposed answer of the registration problem. The validation of registration is measured by the correlation coefficient between two aligned images. In order to evaluate the solution quality, we use a cross-correlation similarity function. Hence, the correlation coefficient method can be used as an objective function which has to be optimized to maximum value. Given two images A and B, the correlation coefficient is defined in Equation (4) as:

(4) $$C(A,B)=\frac{\sum_{i=0}^{N-1}\sum_{j=0}^{N-1}\left(\left(A_{\mathrm{ij}}\bar{-}\bar{A}\right)\times \left(B_{\mathrm{ij}}-\bar{B}\right)\right)}{\sqrt{\sum_{i=0}^{N-1}\sum_{j=0}^{N-1}{\left(A_{ij}-\bar{A}\right)}^{2}\;\times \;\sum_{i=0}^{N-1}\sum_{j=0}^{N-1}{\left(B_{ij}-\bar{B}\right)}^{2}\;}}$$

where *A* and *B* are images of size N × N, while *A_ij_* and *B_ij_* denote pixel values in *A* and *B* respectively. Then the problem is to maximise the correlation function, and for that we select highly fit individuals with higher correlation (fitness) values.

## 6. Proposed Methods

In this work, we have utilized two techniques to accomplish registration –multi-resolution decomposed images in order to reduce the search space and GAs for optimization of the search space. The multi-resolution decomposed image employs two transforms: wavelets and NSCT.

While using the wavelet transform, both the reference image and input or transformed image to be corrected are first decomposed following multi-resolution wavelet decomposition. For each level of decomposition, three images are obtained, namely LL, LH and HL. The sub-band HH includes the high frequency noise which affects image matching, and is, therefore, not useful for registration [28]. The corresponding sub-band couples from the two images are compared using GAs. The purpose is to maximize the correlation coefficient between the two images, in order to find the best parameters of transformation (R,X,Y). At each level of decomposition, the search focuses in the interval around the “best” transformation found at the previous level and is refined at the next level up; working iteratively from the deepest level of decomposition (where the image size is the smallest) to the top level of decomposition, i.e going from coarse to fine spatial resolution. In other words, the parameters found in level *l* are used to estimate the new search space of GAs of level *l-1* with minimizing the population size in order to reduce the search space. The accuracy of this search increases when going from coarse resolution to fine resolution. The registration process terminates when the matching criteria is optimized at the highest resolution level. At fine resolution, we reconstruct the corrected image.

The second multi-resolution decomposition used in this work is the NSCT in which we propose two methods of registration based on this transform. In both ones, we proceed as in the wavelet transform but the NSCT decomposition has more directions or sub-bands.

In the first proposed method, we decompose the two images to be compared into several levels with different directions. The decomposition results are one LL sub-band, which is the approximation of the original image, and different direction sub-bands. At each level of the decomposition, the correlation ratio between corresponding sub-band images of the reference and input images is successively computed and maximized using GAs. We correct the sub-bands of the input image with the optimal transformation parameters (R,X,Y) found during the run process at this level. These obtained parameters are used to refine the search space of lower level. Going from one level to another is done according to two criteria: concentrate the search space around the optimal values found in the previous level, then adapt better the population size by minimising it. This way, the time complexity of the refinement process through different levels is really reduced, while the registration accuracy is increased. This is indeed an adaptable GA. Finally, the corrected image is reconstructed at full resolution with all different corrected sub-bands.

**Figure 4.**
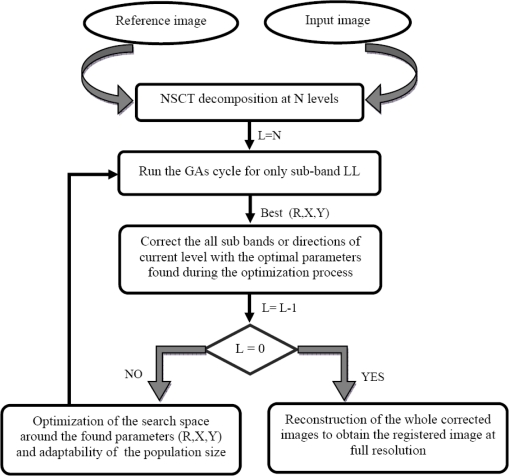
Image registration algorithm based on the second proposed NSCT method.

The results obtained by the first proposed method are better than those of the one based on the wavelet, although the size of the different sub-bands of all levels is the same as that of the original image in the NSCT decomposition because it is a shift-invariant multi-resolution transform; as opposed to the wavelet transform, in which the size of sub-bands decreases with the increase of the level. To overcome this problem, we propose a second method based on the NSCT. This approach consists of performing the GA’s process on only the sub-band LL. At each level of the decomposition, the parameters found with GAs are used to correct the directions or sub-bands of this level. The level changing process is done the same way as that of the dynamic and adaptable one in the first method. At full resolution, we reconstruct the registered image with the corrected sub-band images of all directions. This algorithm is shown in Figure 4.

## 7. Results and Discussion

The GA parameters used in this work are set as follows: the population size in each generation is initially restricted to 80 individuals with a crossover probability of 0.85 and a mutation probability of 0.02, and the algorithm fulfils its generation at a maximum of 100 iterations of processing.

To show the performance of the hybrid GA which combines elitism and fitness sharing techniques, we have first performed our algorithm with a simple GA. For the sharing technique, we have used the parameters sigma = 1.2 and *α* = 1, and for elitism we have conserved 5% individuals of the population at each generation.

**Figure 5.**
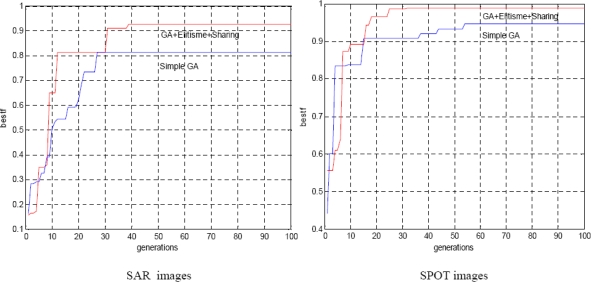
Evolution of best solution during the run of GAs.

Figure 5 illustrates the evolution of the best solution during generations. The parameter ‘bestf’ indicates the fitness of the best individual (maximum fitness) obtained during the run of GAs. It is clear that the higher maximum fitness gives better accuracy in estimating the optimal value, which is indeed obtained by the hybrid GA with no multi-resolution strategy compared to the simple GA. The test algorithm is applied for both SAR and SPOT images.

In this work, for the different level decompositions, the Haar filter has been used for the wavelet transform, while the diamond maxflat filters have been used for the NSCT transform for both directional and pyramidal filters. The wavelet decomposition is carried out up to the third level. As for the NSCT decomposition, we have chosen two levels with four directions each, for both methods. Initially, a population size of 80 has been set for the highest level. When switching from one level to a lower one, this size is reduced by a step of 30 individuals. Thus, at the lowest level, this size will be of only 20 individuals. Moreover, the search space is reduced at each level and is concentrated around the optimal parameter values found in the previous level.

**Figure 6.**
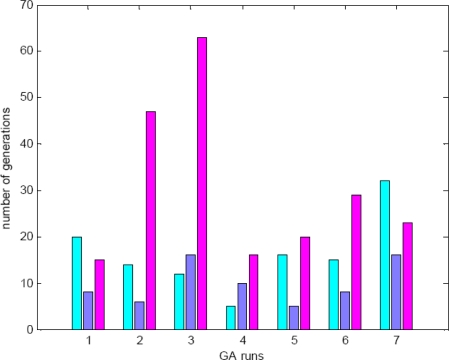
Statistical results for an NSCT2 application.

It is well known that GAs are essentially stochastic search and optimization methods. Results obtained by GAs are only meaningful on a statistical basis since different runs of a GA may lead to different optimal solutions. For a very simple problem, the final optimal solutions obtained may be the same for different runs, but the numbers of generations at which the optimal solutions are obtained could be different. Figure 6 presents the statistical results of the NSCT2 proposed method for an example, in which seven GAs runs have been performed on a case of SPOT images to show the behaviour of these techniques. Two levels of decomposition are used which have lead to three LL sub-bands. For each GA run, the number of generations for levels 0, 1 and 2 are shown from left to right, respectively.

### 7.1. Simulation Results

The experimental tests were performed using different types of satellite images such as SPOT and IKONOS as well as radar images. In these first experiments, the transformed or input images to be corrected are simply the reference images rotated by a 7 degree angle of rotation and displaced by a (13 × 9) pixel translation in the X and Y directions from the center of the reference images. The sensed images are resampled using the bilinear interpolation.

**Figure 7.**
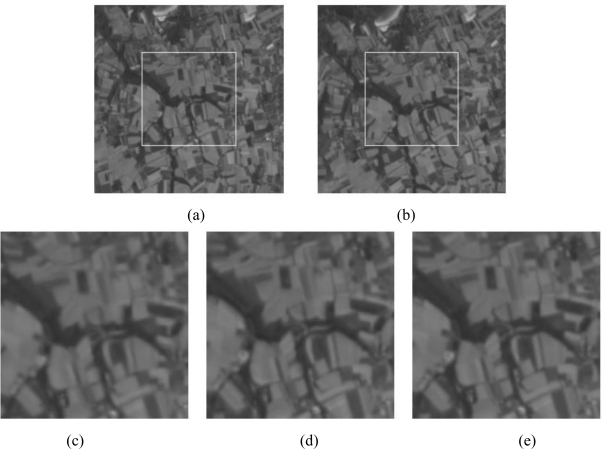
Registration of SPOT images using the three methods: (a) reference image, (b) transformed image to be corrected, (c) registered image using wavelet, (d) registered image using the first proposed method, and (e) the corrected image using the second proposed method.

Figure 7 illustrates a SPOT registered image obtained using the three multi-resolution approaches: wavelet, and the two NSCT methods. The pair of images to be compared is shown with a size of (256 × 256) pixels, in which the region of interest is enclosed in the white box. Thus, the resulting image is of a size of (128 × 128) pixels.

**Figure 8.**
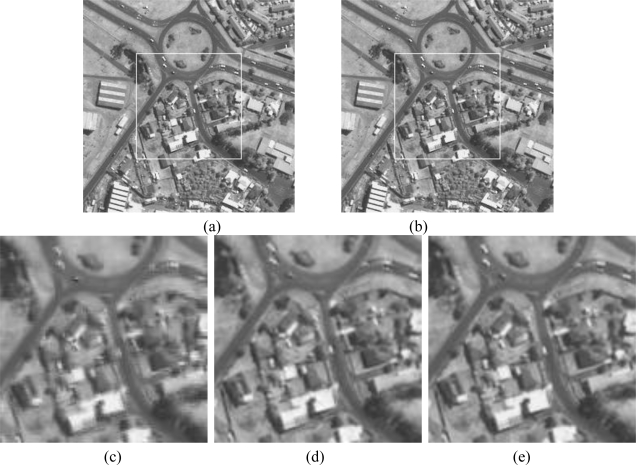
Registration of IKONOS images obtained using the three approaches: (a) the reference image, (b) the transformed image, (c) registered image using wavelet, (d) registered image using the first proposed method, and (e) registered image using the second proposed method.

**Figure 9.**
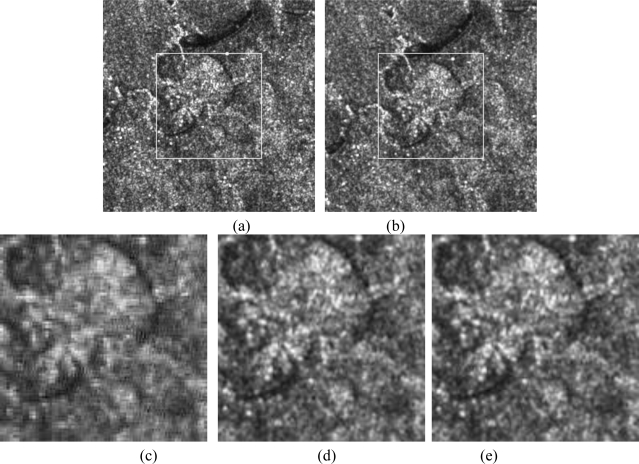
Registration of SAR images (with inherent speckle noise) using the three methods: (a) reference image, (b) transformed image to be corrected, (c) registered image using wavelet, (d) registered image using the first proposed method, and (e) the corrected image using the second proposed method.

We have also tested our registration algorithm on an IKONOS image and a SAR image (with inherent speckle noise), as shown in Figures 8 and 9, respectively.

Moreover, the different analytical results of the registration methods on all image sets are depicted in Table 1. Two measures are considered to determine the accuracy of registration: the correlation coefficient and the root mean square error defined as:

(5) $$\mathrm{RMSE}\;=\;\sqrt{\frac{1}{k}\sum_{i=1}^{k}{\left(P_{i}\;-\;Q_{i}\right)}^{2}}$$

where *P* is the original image and *Q* the corrected one, and *k* is the size of image. In addition, Table 1 shows the computation cost when using an HP Compaq machine, core 2 duo 2.66 GHz CPU.

It is clear from these results that both the NSCT proposed methods perform better in terms of correlation and RMSE than the wavelet method. It is noteworthy that the processing time is lower when performing the NSCT2 method than that of the NSCT1 one. This is due to the fact that we used all sub bands for the first method while we used only the LL sub band for the second one. Moreover, The NSCT2 has been compared to the regular registration method and the results in Table 2 show a significant time processing reduction when using NSCT2.

### 7.2. Real Applications Results

**Figure 10.**
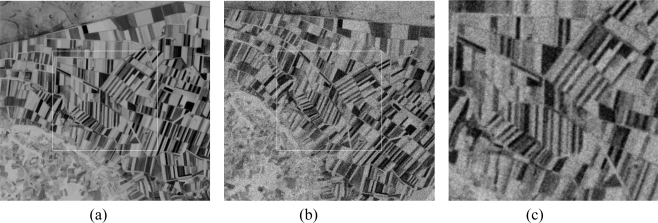
Registration of multi-temporal SPOT images: (a) the input image, (b) the sensed noisy image to be corrected, and (c) the registered image with the second proposed method.

The proposed algorithm works perfectly well in real practical applications as well, even with the presence of noise as shown in Figure 10, which presents the registration results of multi-temporal satellite images. The tested pair of images is one of panchromatic SPOT images acquired at different dates, on one of which we have added a Gaussian white noise. Clearly, the obtained results are really promising. The analytical results obtained with the second proposed method led to an RMSE of 0.0630 and a correlation ratio of about 0.9317.

## 8. Conclusions

Image registration is a very complex problem in the field of image processing. The study of recent multi-resolution search and optimization algorithms applied to image registration has offered new perspectives to handle this challenge. The contribution of this paper is the use of the GAs within a multi-resolution framework based on the NSCT which provides a directional multi-resolution image representation for performing an efficient, robust and accurate rigid registration. Two methods are proposed for registration using GAs. The first one consists of performing the registration for all sub-bands while the second uses just the LL sub band in order to speed up the search and to minimise the time complexity of the optimization process. Both approaches perform better than the wavelet decomposition while the second proposed method is faster than the first one. The simulation results show the effectiveness of this method compared to the other ones. Moreover, this approach, which has been used for registering high resolution satellite and radar images, works perfectly well for multi-temporal images as well, even in the presence of noise.

## Figures and Tables

**Table 1.** Analytical results for the three registration methods.

		**Techniques**		
		**Wavelet**	**NSCT1**	**NSCT2**

**SPOT**	Corr.ratio	0.8298	0.9863	0.9840
	RMSE	0.0462	0.0137	0.0147
	Computation time (s)	929.25	5127.04	1421.20

**IKONOS**	Corr.ratio	0.6935	0.9562	0.9468
	RMSE	0.1245	0.0504	0.0549
	Computation time (s)	1114.73	5213.72	1447.58

**SAR**	Corr.ratio	0.5622	0.9474	0.9294
	RMSE	0.1549	0.0608	0.0703
	Computation time (s)	1065.54	5206.86	1448.27

**Table 2.** Comparison results between the NSCT2 and regular methods.

**Methods**	**Corr. Ratio**	**RMSE**	**Computation time (s)**
**regular**	0.9845	0.0144	167220.00
**NSCT2**	0.9840	0.0147	1421.20

## References

[1] Brown LG (1992). A survey of image registration techniques. ACM Comput. Surv.

[2] Zitovà B, Flusser I (2003). Image registration methods: A survey. Image Vision Comput.

[3] Bentoutou Y, Taleb N, Kpalma K, Ronsin J (2005). An automatic image registration for applications in remote sensing. IEEE Trans. Geosci. Remote Sens.

[4] Taleb N, Bentoutou Y, Deforges O, Taleb A (2001). A 3-D space-time motion evaluation for image registration in digital subtraction angiography. Comput. Med. Imaging Graph.

[5] Bentoutou Y, Taleb N, Chikr el mezouar M, Taleb M, Jetto L (2002). An invariant approach for image registration in digital subtraction angiography. Pattern Recognit.

[6] Bentoutou Y, Taleb N (2005). A 3D space-time motion detection for an invariant approach image registration approach in digital subtraction angiography. Comput. Vision Image Understand.

[7] Bentoutou Y, Taleb N (2005). Automatic extraction of control points for digital subtraction angiography image enhancement. IEEE Trans. Nucl. Sci.

[8] Holland JH (1975). Adaptation in Natural and Artificial System.

[9] Goldberg DE (1989). Genetic Algorithm in Search, Optimization and Machine Learning.

[10] Lobo FG, Goldberg DE Decision Making in a Hybrid Genetic Algorithm.

[11] Silva L, Bellon OR, Gotardo PF, Boyer KL Range image registration using enhanced genetic algorithms.

[12] Mashohor S, Evans JR, Arslan TA Image Registration of Printed Circuit Boards using Hybrid Genetic Algorithm.

[13] LeMoigne J Parallel Registration of Multi Sensor Remotely Sensed Imagery Using Wavelet Coefficients.

[14] Corvi M, Nicchiotti G Multi-resolution Image Registration.

[15] LeMoigne J Towards a Parallel Registration of Multiple Resolution Remote Sensing Data.

[16] LeMoigne J Towards an Intercomparison of Automated Registration Algorithms for Multiple Source Remote Sensing Data.

[17] Pinzon J, Ustin S, Castaneda C, Pierce P Image Registration by Non-Linear Wavelet Compression and Singular Value Decomposition.

[18] Chettri S, Campbell W, LeMoigne J A Scale Space Feature Based Registration Technique for Fusion of Satellite Imagery.

[19] Fonseca L, Manjunath BS, Kenney C Scope and Applications of Translation Invariant Wavelets to Image Registration.

[20] William DR, Karl CW (2003). Wavelet based image registration. Application of digital image processing. SPIE.

[21] Fitzpatrick JM, Grefenstette JJ, Van-Gucht D Image registration by genetic search.

[22] Ozkan M, Fitzpatrick JM, Kawamura K Image Registration for a Transputer-Based Distributed System.

[23] Dasgupta D, McGregor DR (1992). Digital Image Registration Using Structured Genetic Algorithms. Proc. SPIE Int. Soc. Opt. Eng.

[24] Turton B, Arslan T, Horrocks D A hardware architecture for a parallel genetic algorithm for image registration.

[25] Ou G, Chen H, Wang W (1996). Real-time image registration based on genetic algorithms. Proc. SPIE.

[26] Maslov IV, Gertner I Gradient-based genetic algorithms in image registration.

[27] Laksanapanai B, Withayachumnankul W, Pintavirooj C, Tosranon P (2006). Acceleration of genetic algorithm with parallel processing with application in medical image registration.

[28] Chalermwat P, El-Ghazawi T Multi-resolution image registration using genetics.

[29] Chalermwat P, El-Ghazawi T, LeMoigne J (1999). A 2-phased GA-based Image Registration on Parallel Clusters. IPPS/SPDP’99 Workshops Parallel and Distributed Processing.

[30] Serief C, Barkat M, Bentoutou Y, Benslam M (2009). Robust feature points extraction for image registration based on the nonsubsampled contourlet transform. Int. J. Elec. Commun.

[31] Mallat SG (1993). A Theory for Multi-resolution Signal Approach. J. Photogramm. Remote Sens.

[32] Do MN, Vetterli M (2005). The contourlet transform: An efficient directional multiresolution image representation. IEEE Trans. Image Process.

[33] Simoncelli EP, Freeman WT, Adelson EH, Heege DJ (1992). Shiftable multiscale transforms. IEEE Trans. Inform. Theory.

[34] da Cunha AL, Zhou JP, Do MD (2006). The nansubsampled contourlet transform: Theory, design, and applications. IEEE Trans. Image Process.

[35] Giuseppe P, Luigi T A Niche Based Genetic Algorithm for Image Registration.

[36] Gao L, Hu YW (2006). Multi-target matching based on Niching genetic algorithm. Int. J. Comput. Sci. Netw. Security.

